# A65 PREDICTING TRANSITION SUCCESS IN YOUNG ADULTS WITH INFLAMMATORY BOWEL DISEASE: PRELIMINARY RESULTS

**DOI:** 10.1093/jcag/gwab049.064

**Published:** 2022-02-21

**Authors:** A Bihari, K Goodman, E Wine, K Kroeker

**Affiliations:** 1 University of Alberta, Edmonton, AB, Canada; 2 Pediatrics, University of Alberta, Edmonton, AB, Canada

## Abstract

**Background:**

Patients diagnosed with inflammatory bowel disease (IBD) in childhood present more often with extensive disease, are more likely to be admitted to hospital and are less adherent with clinic appointments. Due to these risks, a smooth, uninterrupted transition from pediatric to adult care should be a priority. We have conducted interviews with providers, patients, and parents about their opinions on indicators of successful transition. Themes of successful transition that emerged included independence in seeking care and disease management. Characterizing successful transition based on stakeholder input makes it possible to monitor its achievement and identify its determinants.

**Aims:**

This study aims to: 1) describe the frequency of success indicators in transitioned patients and 2) identify predictors associated with success indicators. We hypothesize that patients with more experience in pediatric care (e.g., younger age at diagnosis or on biologics) are more likely to achieve success.

**Methods:**

We conducted a retrospective medical chart review to obtain data on patients who transitioned to adult care between January, 2014 - September, 2019 at the University of Alberta. We abstracted potential predictors, including social and disease factors, at first adult appointment which had notes on pediatric history. We chose available success indicators related to two themes: independence in seeking care (e.g., attending appointments, communicating for oneself) and disease management (e.g., lab work frequency and medication adherence). We abstracted selected success indicators within a two-year period from first appointment in adult care. We used Poisson and logistic regression to estimate incidence rate ratios (IR) and odds ratios (OR) for the association of potential predictors with success indicators.

**Results:**

We reviewed medical charts of 99 patients. At first adult appointment, the median age at diagnosis was 14.5 years old (IQR: 13.2 – 15.9) and 57.6% of patients were on biologic agents. Within two years, 42.4% of patients required a change to a different therapy, 22.2% had at least two instances where a parent called on their behalf, and 16.2% had notes of medication nonadherence in adult care. Regression analysis (Table 1) estimated that patients who lived > 100km from clinic had a lab work incidence rate in the first year that was two-thirds that of patients who lived closer. Strong predictors of non-adherence in adult care included chart notes on pediatric medication non-adherence (OR~12) and, inversely, taking biologics (OR=0.34).

**Conclusions:**

These results identified factors that could be used to identify patients likely to have poor outcomes following transition to adult care. These are preliminary results; we plan to analyze a total of 350 medical charts.

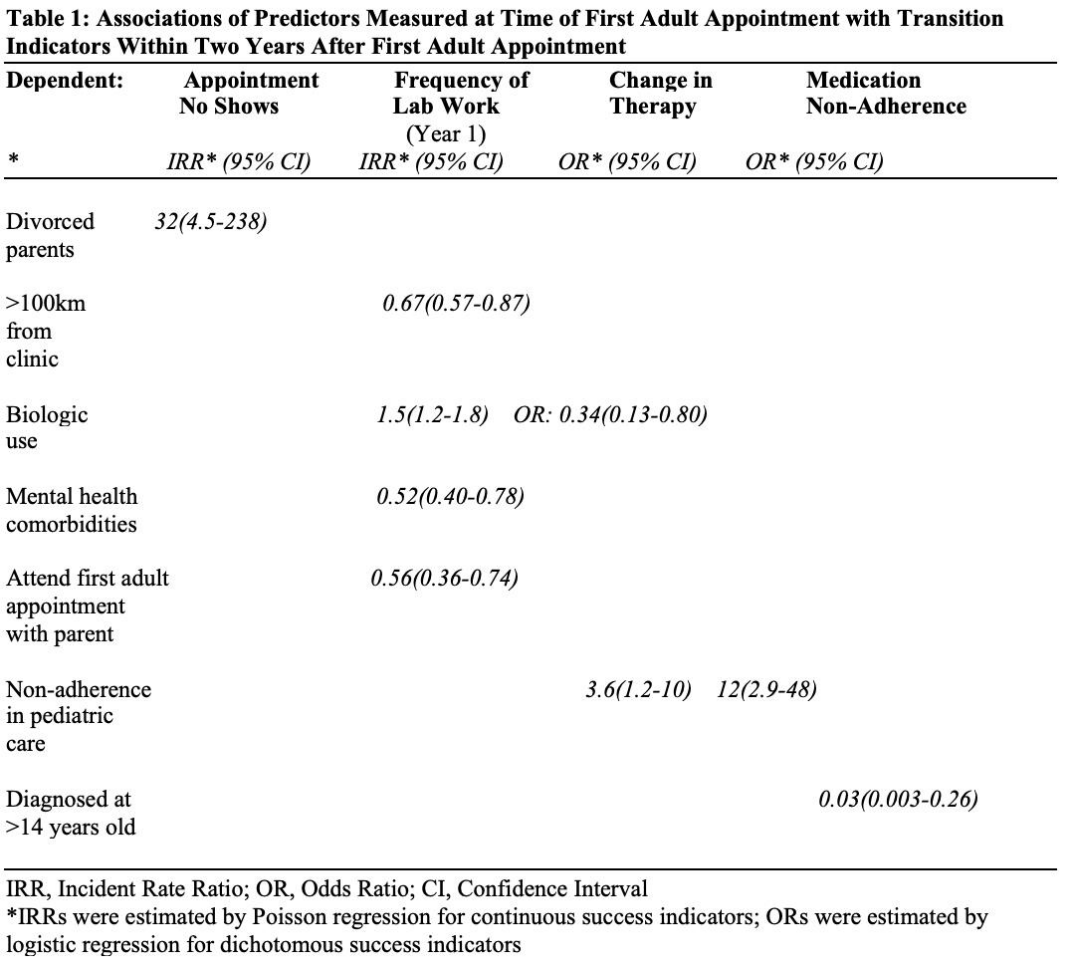

**Funding Agencies:**

None

